# Clinical spectrum of Celiac disease from a cardiology perspective

**DOI:** 10.12669/pjms.38.4.6129

**Published:** 2022

**Authors:** Gard M. S. Myrmel, Torbjørn Lunde, Sahrai Saeed

**Affiliations:** 1 Gard M. S. Myrmel, Department of Heart Disease, Haukeland University Hospital, Bergen, Norway; 2 Torbjørn Lunde, Department of Heart Disease, Haukeland University Hospital, Bergen, Norway; 3 Sahrai Saeed, Department of Heart Disease, Haukeland University Hospital, Bergen, Norway

**Keywords:** Celiac disease, Echocardiography, Extra-intestinal manifestations, Myocarditis

Celiac disease (CD) is a common immune-mediated inflammatory disease with a global prevalence estimated at 0.6-1.0 percent.^1^ However, the true prevalence is suspected to be much higher as demonstrated in serological screening studies.^2^ CD is caused by ingestion of dietary gluten and related proteins in genetically predisposed individuals. It mainly affects the small intestine, but extra-intestinal manifestations have been described in numerous organs including the central nervous system, skin, liver and joints.^3^ CD may present with a wide range of symptoms and signs such as gastrointestinal symptoms (abdominal pain, diarrhea and weight loss) and signs of malabsorption including iron deficiency anemia and Vitamin-D deficiency, that may ultimately lead to osteoporosis and pathological fractures. The condition is normally treated with a strict gluten-free diet (GFD), which may be difficult to adhere to, and even small amounts of gluten (10-50 mg per day) may cause damage to the intestinal mucosa. New therapeutic approaches including oral transglutaminase 2 inhibitors have recently demonstrated promising results,^4^ and may become important alternatives to GFD in the future, although none of them are currently approved for clinical use.

In a recent edition of the journal, Arshad et al. presented results from a retrospective cross-sectional study of 126 adult patients (62% females, mean age 35.5 years) with CD.^5^ The study confirmed that vague symptoms like fatigue (24%), abdominal pain (56%), elevation of liver enzymes and signs of malabsorption like microcytic anemia (36.5%) were the most common, and often the only clinical findings in patients with CD, which should prompt further investigation with serological testing and endoscopy. CD has previously been described as a disease of mainly people by European ancestry with a reported prevalence in Asian countries of 0.5%. However, as the authors point out, there is reason to believe that the prevalence is substantially higher than previously assumed. The current study is important since it provides an important insight to the epidemiology, clinical presentation and treatment of CD in Pakistan. The authors report that more than half of the study population was either lost to follow-up or non-compliant, highlighting the need for better availability and lower cost of GFD, which may facilitate better patient adherence to GFD.

Furthermore, it was reported that psychiatric disturbances were found in 12.7%. This included depression and anxiety, which are major reasons for non-compliance often necessitating a multidisciplinary approach and follow-up. Despite CD being considered as a condition largely affecting the small intestine, cardiovascular manifestations may occur by virtue of its immune-mediated pathogenesis. In particular, myocarditis that is a rare complication of CD (6-8) and should be suspected when patients present with cardiac symptoms or ECG abnormalities (Table-I). CD has an estimated prevalence between 1.8 – 5.7 % in patients with myocarditis, which is markedly higher than in the general population. In a study by Frustachi *et al*. a total of 187 patients with myocarditis were screened, and CD was confirmed by biopsy in 4.4%.^6^ Furthermore, Sategna-Guidetti *et al*. demonstrated IgA serum antibodies directed against myocardium in patients with untreated CD, that was not found in patients with treated CD, or in healthy controls.^7^ This supports a pathophysiological link between CD and autoimmune myocarditis. When diagnosing myocarditis, echocardiography is the first-line imaging modality (Table-I, Fig.1), but cardiac magnetic resonance (CMR) imaging is often necessary to confirm the diagnosis. In selected cases, there may also be need for myocardial biopsy. A swift diagnosis and implementation of GFD and/or immunosuppressive therapy is necessary to avoid progression into feared complications like dilated cardiomyopathy, heart failure and malignant cardiac arrhythmias. Conversely, CD should be suspected in patients with myocarditis when symptoms of CD is present, particularly iron deficiency anemia, as demonstrated in a recent case report^8^ of a young man with probable CD related myocarditis. The patient presented to the hospital after three months of dyspnea, chest pain and palpitations, and after extensive investigations, a diagnosis of myocarditis was made. The incidental finding of iron deficiency anemia led to further investigation including serological testing and endoscopic biopsies confirming CD, after which a GFD was commenced. At 6-month follow-up, a CMR imaging did not show any signs of ongoing inflammation and the ejection fraction had increased from 25-35%, demonstrating the importance of identifying CD in patients with myocarditis and implementing GFD. However, given the high prevalence of both disorders (CD and myocarditis), their coexistence, independently of each other, is not impossible.

**Table-I:** Examples of ECG and echocardiography findings in myocarditis.

***ECG findings***
***ST- segment and T-wave abnormalities:*** ST-elevation, ST-depression, T-wave inversion
***Arrhythmias:*** I to III degree atrioventricular-block, ventricular tachycardia or fibrillation, sinus arrest, frequent premature beats, supraventricular arrhythmias.
***Other:*** Abnormal q-waves, low voltage, new bundle branch block
***Echocardiography findings***
***Functional abnormalities:*** RV (reduced TAPSE and RV S’) and LV systolic or diastolic dysfunction. New LV or RV regional wall motion abnormalities.
***Structural abnormalities:*** Increased RV and LV volumes. Increased wall thicknesses.
***Other:*** Pericardial effusion. Intracardiac thrombus. Significant functional tricuspid and mitral regurgitations.

RV, Right ventricle; LV, Left ventricle; TAPSE, Tricuspid annular plane systolic excursion.

**Fig.1:**
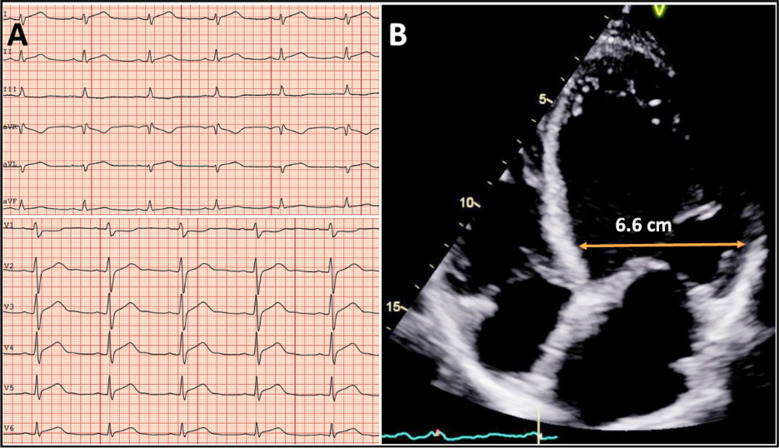
Electrocardiogram and echocardiography images of two different patients with myocarditis. Electrocardiogram (A) shows sinus rhythm and global ST-elevations in a patient with myocarditis. Echocardiography image of another patient (young male with CD); Apical 4-chamber view demonstrates a dilated left ventricle of 6.6 cm (B).

## CONCLUSION

The work of Arshad *et al*. provides important insights on the epidemiology, clinical findings and treatment of patients with CD in Pakistani adults, filling some important knowledge gaps, and highlighting the importance of timely diagnosis and the need for better access and lower cost of gluten-free foods in low-middle income areas. However, as highlighted by the authors, the small sample size, retrospective design, lower proportion of patients with repeated endoscopy and higher proportion of patients lost to follow-up were the study limitations. Hence, larger prospective follow-up studies are warranted in the future. Severe extra-intestinal complications like myocarditis and neurological disturbances are not as uncommon as previously thought.

### Key educational messages:

- Autoimmune myocarditis is a rare complication in CD, and should be kept in mind when patients present with symptoms of heart failure, arrhythmia and chest pain.
- Reversely, CD should be also considered as a differential diagnosis in patients with myocarditis and gastrointestinal symptoms or unexplained anemia.

### Authors’ Contribution:

**GMSM** wrote the first draft of the article which was subsequently revised by **TL and SS.** All authors approved the final submission.
